# ﻿*Dickyyuellaargentinensis* a tentative new genus and species of Cardiochilinae (Hymenoptera, Braconidae) from the Neotropical region

**DOI:** 10.3897/zookeys.1208.128640

**Published:** 2024-07-29

**Authors:** Ilgoo Kang, Michael J. Sharkey

**Affiliations:** 1 Department of Entomology, Kyungpook National University, Sangju, Gyeongsangbuk-do, Republic of Korea Kyungpook National University Sangju Republic of Korea; 2 The Hymenoptera Institute, Redlands, CA, USA The Hymenoptera Institute Redlands United States of America

**Keywords:** Argentina, Ichneumonoidea, morphology, Neotropics, new species, non-cyclostomes, parasitoid, taxonomy, wasp

## Abstract

*Dickyyuella* Kang & Sharkey, **gen. nov.** is a novel addition to the microgastroid complex of Braconidae. Taxonomic assignment within this complex posed challenges initially due to the presence of putatively plesiomorphic characters. However, closer examination revealed affiliations with the microgastroid complex, supported by morphological features such as the location of spiracles on the first metasomal tergum and the absence of spiracles on the seventh metasomal tergum. Based on the following two morphological characters, the presence of an inverted Y-shaped groove on the first metasomal tergum and pectinate tarsal claws, *Dickyyuella* Kang & Sharkey, **gen. nov.** is tentatively placed within Cardiochilinae Ashmead, 1900 despite uncertainties surrounding phylogenetic relationships. This article provides the diagnosis of *Dickyyuella* Kang & Sharkey, **gen. nov.**, the description of *Dickyyuellaargentinensis* Kang & Sharkey, **sp. nov.**, and a discussion of the taxonomic placement of the new genus within the microgastroid complex.

## ﻿Introduction

Members of Braconidae Latreille, 1829 are traditionally divided into two major groups, cyclostomes and non-cyclostomes, depending on the presence/absence of an opening between the clypeus and mandibles (Sharkey 1993). Non-cyclostome members lack this opening, making the labrum mostly invisible. Among the non-cyclostome subfamilies, there is a recently derived group, the microgastroid complex, which comprises seven subfamilies: Cardiochilinae Ashmead, 1900, Cheloninae Föerster, 1863, Dirrhopinae van Achterberg, 1984, Khiokhoiinae Mason, 1983, Mendesellinae Whitfield & Mason, 1994, Microgastrinae Föerster, 1863 and Miracinae Viereck, 1918. All members of the microgastroid complex are known as endoparasitoids of Lepidoptera (Yu et al. 2016). The phylogenetic relationships among the subfamilies of this complex have garnered significant attention from braconidologists, who have attempted to resolve these relationships using morphological characteristics, molecular data, and polydnaviruses (Quicke and van Achterberg 1990; Whitfield and Mason 1994; Whitfield 1997; Dowton and Austin 1998; Murphy et al. 2008). Recent studies based on ultraconserved elements (UCEs) data have resolved Dirrhopinae as a sister taxon to Cheloninae, thereby confirming its placement in the microgastroid complex (Jasso-Martínez et al. 2023).

While examining specimens in the Entomology Research Museum at the University of California, Riverside (UCRC; Riverside, CA, USA), the second author discovered a highly distinctive braconid specimen from the Neotropical region and shared this discovery with a few other braconid experts. Following examinations by each author, we initially hypothesized that the specimen might represent a new braconid subfamily. However, further analysis led us to describe a tentative new genus of Cardiochilinae, *Dickyyuella* Kang & Sharkey, gen. nov., and a new species *Dickyyuellaargentinensis* Kang & Sharkey, sp. nov. based solely on a single specimen. This specimen shares some characteristics with other subfamilies of the microgastroid complex and is distinct from the other genera within Cardiochilinae.

## ﻿Material and methods

The singleton specimen was borrowed from UCRC and examined by both authors. Leica MZ 16 and MZ75 stereomicroscopes were used to the examine specimen. Images of the specimen were taken using a JVC digital camera mounted on the Leica MZ 16 microscope and were stacked using Automontage software (Syncroscopy). The stacked images were then edited using Adobe Photoshop^®^ CS 6 and Photoshop^®^ CC 2024 v. 25.7.0 (Adobe Systems, Inc.). Terms for external morphology and wing venation are based on Sharkey and Wharton (1997). Terms for external sculptures follow Harris (1979). The following are acronyms used in this article except abstract: T1: first metasomal tergum; T2: second metasomal tergum; T7: seventh metasomal tergum. Morphometric characters were measured using Adobe Photoshop^®^ CC 2024 v. 25.7.0. All measurements are provided in millimeters, with numbers in parentheses in the species description representing the actual size of each body part.

## ﻿Results and discussion

### ﻿Taxonomy

#### ﻿ Dickyyuella

Taxon classificationAnimalia

Kang & Sharkey gen. nov.

64CD980A-2F73-57AF-A784-CD853C13CB04

https://zoobank.org/5FC120A3-326D-423A-8913-3A1C8B673D5B

Fig. 1A–E

##### Type species.

*Dickyyuellaargentinensis* Kang & Sharkey, sp. nov.

##### Diagnosis.

**Figure 1.**
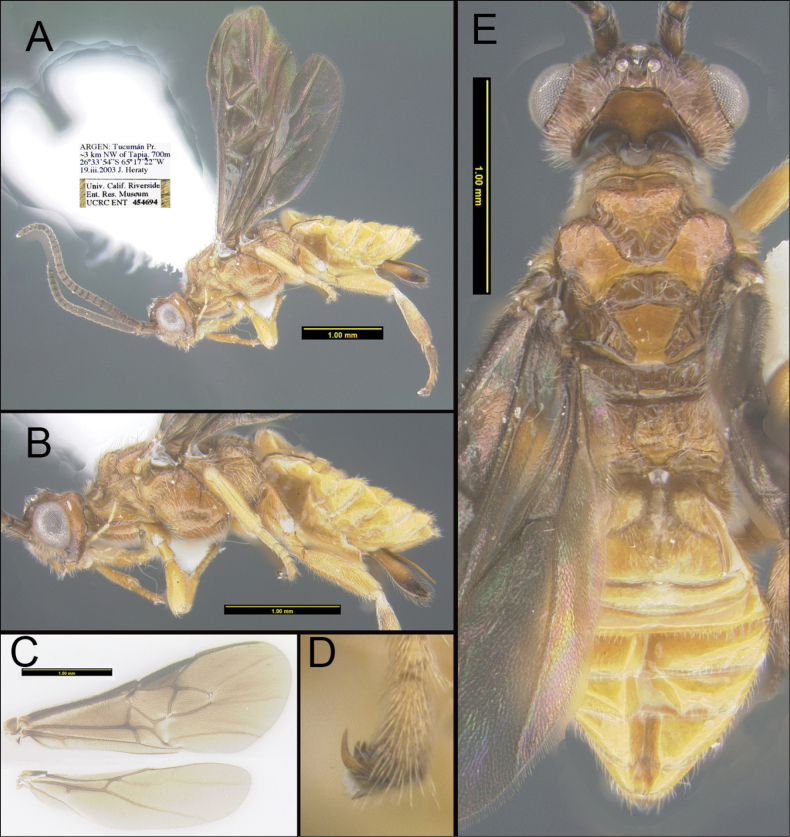
*Dickyyuellaargentinensis* Kang & Sharkey, sp. nov. holotype **A** lateral habitus of the specimen, including antennae and wings **B** lateral view, zoomed in on head, mesosoma, and metasoma **C** wings **D** hind tarsal claw **E** dorsal habitus of the specimen, zoomed in on head, mesosoma, and metasoma.

Body relatively small compared to members of the other cardiochiline genera, with strong sculpture, especially on mesosoma. Antenna thick (Fig. 1A). Eyes bare (Fig. 1B). Occipital carina well developed dorsally, absent ventrally (Fig. 1E). Most of head with weak microsculpture. Median ocellus surrounded medially and laterally by a smooth, curved ridge. Pronotum bilobed anteriorly with a transverse plate dorsally. Notauli deeply impressed and entirely costate (Fig. 1E). Median lobe of mesoscutum bilobed. Scutellar sulcus deep with a median carina (Fig. 1E). Scutellum smooth and flat. Postscutellar depression absent (Fig. 1E). Epicnemial carina strong and complete (Fig. 1B). Precoxal sulcus well defined with ~5 costulae (Fig. 1B). Propodeum rugose with a large, well-defined median areola. Apical abscissa of RS entirely nebulous and almost straight, very slightly curved posteriorly (Fig. 1C). (RS+M)b about 3 × longer than m-cu vein (Fig. 1C). 1M about 3 × longer than m-cu vein; lacking distinct claval lobe. Veins M+Cu and M about equal in length. Hind basitarsomere swollen (Fig. 1A). Tarsal claws rather large with pectinate base (Fig. 1D). Spiracle of T1 on membranous laterotergite (Fig. 1B). T1 wide with carinate lateral margins; medial area of T1 with an inverse Y-shaped depression (Fig. 1E).

##### Biology.

Unknown.

##### Distribution.

Neotropics.

##### Etymology.

The genus name is a patronym in honor of Dicky Sick Ki Yu, who developed Taxapad and made significant contributions to Braconidae and Ichneumonidae systematics research. Gender is feminine.

##### Notes.

The members of *Dickyyuella* Kang & Sharkey, gen. nov. will run to couplet 1 in the key to the world genera by Dangerfield et al. (1999), but it can be easily distinguished from members of *Heteropteron* and *Neocardiochiles* by the size, well-developed occipital carina, deep and broad notauli, large median areola on propodeum, and rugose propodeum.

###### ﻿Species description

#### ﻿ Dickyyuella argentinensis

Taxon classificationAnimalia

Kang & Sharkey sp. nov.

4F2C09B2-45EA-582D-97EE-924BCB139AE8

https://zoobank.org/C178D89C-B759-4E20-BD20-65946C833E4E

##### Material examined.

***Holotype*. Argentina** • ♀, Tucumán Pr., ~3 km NW of Tapia; 700 m, 26°33′54″S, 65°17′22″W; 19.iii.2003; J. Heraty. Will be housed in UCRC.

##### Description.

***Body length***: 3.7 mm. ***Length of forewing***: 3.3 mm. ***Length of hind wing***: 2.6 mm. ***Head*.** Antenna relatively thick with 24 flagellomeres; first flagellomere 1.5 × as long as second. Median width of eye 0.3 × longer than median width of gena in lateral view (0.3:0.1). Width of anterior ocellus 0.9 × longer than distance between posterior ocelli (0.08:0.09). Apex of clypeus convex with a smooth apical margin. Maxillary palpus 5-segmented; labial palpus 4-segmented. Occipital carina well developed dorsally, absent ventrally (This may be a pseudo-occipital carina, i.e., secondarily derived, as is found in some Agathidinae, e.g., *Marjoriella* spp.). Most of head with weak microsculpture contrasting sharply with the smooth, glabrous occiput. Malar suture present. Interantennal space with a bicarinate ridge. Median ocellus surrounded medially and laterally by a smooth, curved ridge. ***Mesosoma*.** Pronotum bilobed anteriorly with a transverse plate dorsally. Notauli deeply impressed and entirely costate. Median lobe of mesoscutum bilobed. Scutellar sulcus deep with a median carina; median width of scutellar sulcus 0.4 mm; median length of scutellar sulcus 0.1 mm; median length of scutellar sulcus 0.1 × longer than median length of mesosoma in dorsal view (0.1:0.9). Scutellum smooth and flat. Postscutellar depression absent. Propleuron lacking a posterolateral lobe. Epicnemial carina strong and complete. Precoxal sulcus well defined with ~5 costulae. Propodeum rugose with a large, well-defined median areola. ***Wings*.** Forewing M+Cu entirely tubular; 1RS vein long; second submarginal cell large and greatly compressed apically, trapezoid, maximum length of the cell 1.6 × longer than its maximum height (0.46:0.28); apical abscissa of RS entirely nebulous and almost straight, very slightly curved posteriorly; (RS+M)b about 3 × longer than m-cu vein; 1M about 3 × longer than m-cu vein; anal crossvein indicated by a slight swelling on vein A. Hind wing unremarkable; lacking distinct claval lobe; veins M+Cu and M about equal in length; r crossvein absent. ***Legs*.** Midtibia 3.4 × longer than midbasitarsomere (0.64: 0.19). Hind femur 0.9 × longer than hind tibia (0.75:0.84). Hind basitarsomere swollen. Tarsal claws rather large with pectinate base. ***Metasoma*.** Metasoma 1.1 × longer than mesosoma (1.70:1.56). Spiracle of T1 on membranous laterotergite. T1 0.7 × longer than its apical width (0.63:0.45), with carinate lateral margins; median area of T1 with an inverse Y-shaped depression. Remaining terga smooth and rather weakly sclerotized. T2 transverse, much wider than long. Hypopygium acute apically and not nearly reaching apex of metasoma. Ovipositor sheath about half as long as metasoma, strongly compressed laterally, with fine sparse setae. Ovipositor simple, slightly downcurved but otherwise unmodified. ***Color*.** Head and mesosoma mostly light brown; antenna brown, foreleg and midleg entirely pale, hind tibia and tarsus yellow medially, hind claw brown. Metasoma mostly pale except ovipositor sheath, ovipositor sheath light brown basally, apically dark brown. Wings entirely infuscate.

**Male.** Unknown.

##### Biology.

Unknown.

##### Distribution.

Neotropics. *Dickyyuellaargentinensis* Kang & Sharkey, sp. nov., is known from Tapia, Tucumán Pr., Argentina, near Rio India Muerta.

##### Etymology.

The species is named after the collecting country, “Argentina”.

### ﻿Taxonomic placement

**Figure 2.**
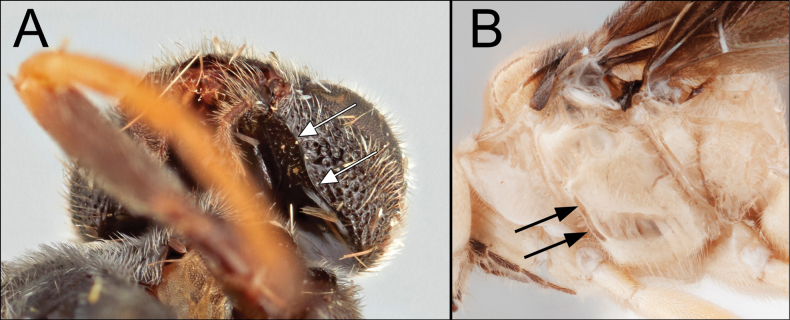
**A** Posteroventral head of *Austerocardiochiles* sp.; arrows: occipital carina **B** mesopleuron of *Bohayellarodrigodiazi* Kang, 2022; arrows: epicnemial carina.

*Dickyyuella* Kang & Sharkey, gen. nov., is tentatively placed as a new member of the microgastroid complex. This is based on three synapomorphies, i.e., spiracle of T1 on the laterotergite; spiracle of T7 absent; apical abscissa of forewing vein RS nebulous (not tubular). We had some difficulty placing this species phylogenetically within the microgastroid complex due to the presence of what are usually considered plesiomorphic characters, based on Cheloninae as the outgroup (Whitfield and Mason 1994; Whitfield 1997; Belshaw et al. 1998; Dowton and Austin 1998; Dowton et al. 1998; Dowton et al. 2002; Banks and Whitfield 2006; Murphy et al. 2008; Sharanowski et al. 2011), i.e., complete occipital and epicnemial carinae. Since the occipital and epicnemial carinae are rarely present in the microgastroid complex and partially developed in a few scattered taxa, we consider the condition to be secondarily derived states. The former character state is rarely known in the complex except for a few species of Microgastrinae, e.g., *Philoplitis* Nixon, 1965 (Ranjith et al. 2019). Some cardiochiline members, e.g., *Austerocardiochiles* Dangerfield, Austin & Whitfield, 1999 and *Psilommiscus* Enderlein, 1912, have partially developed occipital carina in the malar region (Fig. 2A). The epicnemial carina is rare, being found in a few Microgastrinae, e.g., *Fornicia* Brullé, 1846 and *Snellenius* Westwood, 1882 some members of Cardiochilinae, e.g., *Austerocardiochiles*, *Bohayella* Belokobylskij, 1987 and *Toxoneuron* Say, 1836 (Fig. 2B), and weaker in *Mendesella* Whitfield & Mason, 1994 of the Mendesellinae (Whitfield, pers. comm. 2021). In Microgastrinae, the condition does not seem to be the ground-plan state for the subfamily. Of the two genera in Mendesellinae, only species of *Mendesella* have an epicnemial carina, so the ground-plan of the subfamily is equivocal. Based on these two apparently plesiomorphic character states, our first impression of the specimen was that it may be a new subfamily, sister to the remaining microgastroids, who possess apomorphic states of these characters.

Closer inspection of the specimen revealed that the first metasomal median tergite has an inverted Y-shaped groove, which is a unique character state within the microgastroids and possessed only by members of Cardiochilinae (Fig. 1E). Further evidence to suggest membership is the pectinate tarsal claws which are found in the majority of cardiochiline genera and are relatively rare in other microgastroids, e.g., a few species of *Apanteles* Föerster, 1863, *Carlmuesebeckius* Fernandez-Triana, 2018, *Ohenri* Fernandez-Triana, 2018 (Fernandez-Triana and Boudreault 2018) (Fig. 1D). The phylogenetic relationships of Cardiochilinae are largely conjecture, despite the best efforts of Dangerfield et al. (1999); therefore, it is unclear if this is ground-plan or derived. Based on these ambiguous phylogenetic cues we favor the Cardiochilinae hypothesis. This implies that the occipital and epicnemial carinae are secondarily derived and there is no reason to believe that *Dickyyuella* Kang & Sharkey, gen. nov. is the sister to all other Cardiochilinae, although there is no evidence to the contrary either.

## Supplementary Material

XML Treatment for Dickyyuella

XML Treatment for Dickyyuella argentinensis

## ﻿Additional information

### Author ORCIDs

Ilgoo Kang https://orcid.org/0000-0002-8501-1758

Michael J. Sharkey https://orcid.org/0000-0001-6201-7340

### Data availability

All of the data that support the findings of this study are available in the main text.
